# Editorial: Case reports in cancer imaging and image-directed interventions : 2022

**DOI:** 10.3389/fonc.2023.1310454

**Published:** 2023-11-21

**Authors:** Antonio Bottari

**Affiliations:** Dipartimento di Scienze Biomediche, Odontoiatriche, Morfologiche e Funzionali per Immagini, Università degli Studi di Messina, Messina, Italy

**Keywords:** interventional radiology (IR), interventional oncology (IO), cancer imaging, image- guided biopsies, image-guided radiotherapy

Today, imaging is an indispensable tool for cancer detection and characterization.

Both morphological and functional imaging, independently but, above all, in an integrated manner, allow early diagnosis of numerous neoplastic pathologies.

In recent years, technology has enabled notable growth in diagnostic imaging thanks, for example, to the possibility of acquiring very thin slices, new MRI sequences, or increasingly elaborate and high-performance postprocessing, which is extremely useful in some pathologies or in certain anatomical districts (1, 2).

Nowadays, besides the diagnostic role, there is the possibility of using imaging as a guide to treat some of these pathologies in a less invasive way.

Interventional radiology and, specifically, interventional oncology have by now a consolidated role in the therapeutic process of many neoplasms, assuming the role of the fourth pillar of anti-tumor therapy alongside surgery, radiotherapy, and medical oncology (3-7).

In this Frontiers Research Topic, the authors were expected to provide case reports that highlight unique cases of patients that present with an unexpected diagnosis, treatment outcome, or clinical course (8).

Several contributions focused on the diagnosis of rare tumors or rare manifestations of more common tumors (Li et al.; Wang et al.; Hu et al.; Fischerova et al.).

Li et al. presented a case of osteofibrous dysplasia-like adamantinoma, a rare bone tumor (0.4% of all primary bone tumors) recently proposed in the new WHO classification of bone tumors of 2020 and classified as an intermediate locally aggressive tumor. Imaging can demonstrate the lesion and could provide support for differential diagnosis, although a multidisciplinary evaluation is required to reach the diagnosis.

Another very rare tumor that could involve bone was reported by (Wang et al.).

Proliferating trichilemmal tumors originate from the outer root sheath of hair follicles generally located in the head and neck region. Other localizations are uncommon in this condition.^18^ F-FDG-PET could be very useful in identifying both the tumor and any metastatic lymph nodes.

Starting from the presentation of a case, Hu et al. provide a comprehensive literature review of primary intracranial Ewing sarcomas/primitive neuroectodermal tumors, an extremely rare condition with poor prognosis. Unfortunately, only in few cases, they manifest themselves with relatively specific features at imaging; therefore, final diagnosis mainly depends on pathology and immunohistochemistry.

Fischerova et al. reported a rare case of retroperitoneal nodal endometroid carcinoma diagnosed through US-guided biopsy without any macroscopic relevance in the abdomen and pelvis. Immunohistochemical and genetic analysis helped to detect an unknown Lynch syndrome, a congenital condition that could lead to carcinogenesis.

Another aspect of the present Research Topic has been reported by Zhang et al., Li et al., and (Zhang et al.). All of them focused their articles on the usefulness of the uncommon application of specific imaging techniques in certain clinical conditions.

Zhang et al. proposed the use of multimodality US in the detection and characterization of metastasis of rare subtype of renal cell carcinoma (fumarate hydratase-deficient RCC). Application of grayscale, color Doppler, power Doppler, and contrast-enhanced US allow to detect metastasis; characterization could be feasible by ultrasonographic findings accompanied by US-guided biopsy.

In their article, Li et al. enhanced the strength of ^68^Ga-conjugated fibroblast-activation protein inhibitor (FAPI) PET/CT versus ^18^F-FDG in the identification process of intra-abdominal breast cancer metastasis thanks to a better tumor-to-background ratio and lack of interference by non-specific gastrointestinal physiological uptake.

Zhang et al. described contrast-enhanced ultrasound (CEUS) findings of intraperitoneal nodal large B-cell lymphoma. Usually, CT and PET/CT are employed for diagnosis and staging, but in recent years, many studies have focused on alternative imaging methods. CEUS can demonstrate the changes of vascularity of lymph nodes and, at the same time, allow to perform US-guided biopsy to confirm diagnosis.

This Research Topic is completed by two Case Reports involving hepatic diseases.

Shao et al. described a unique imaging pattern of a metastatic neuroendocrine neoplasm characterized by a steatosis area surrounding the hepatic localization. In this article, US findings were reported, and the support of CEUS in differential diagnosis was enhanced.

Congenital portosystemic shunts in children were the focus of (Zhang et al.). The prevalence of these vascular anomalies is 1 in 30000-50000 neonates and may present with various symptoms.

Imaging is essential in diagnosis with US as the first modality of choice in early identification, allowing the prompt management of potentially life-threatening manifestations. Moreover, imaging has a strong role in treatment thanks to the efficacy of endovascular techniques in occluding the shunts.

In conclusion, the contributions presented in this Research Topic have enriched our knowledge of cancer imaging, shedding light on rare neoplastic conditions, their imaging findings, and the implications for the choice of appropriate treatment, often performed under imaging guidance.

All of these studies provide suggestions for further investigation in this field.

## Author contributions

AB: Writing – original draft, Writing – review & editing.

